# Pachymic Acid Attenuated Doxorubicin-Induced Heart Failure by Suppressing miR-24 and Preserving Cardiac Junctophilin-2 in Rats

**DOI:** 10.3390/ijms221910710

**Published:** 2021-10-02

**Authors:** Nahla N. Younis, Alaa Salama, Mohamed A. Shaheen, Rana G. Eissa

**Affiliations:** 1 Biochemistry Department, Faculty of Pharmacy, Zagazig University, Zagazig 44519, Egypt; ranaeissa@hotmail.com; 2 Cardiology Department, Faculty of Human Medicine, Zagazig University, Zagazig 44519, Egypt; salamaalaa137@gmail.com; 3 Histology and Cell Biology Department, Faculty of Human Medicine, Zagazig University, Zagazig 44519, Egypt; drmohamedshaheen@yahoo.com

**Keywords:** heart failure, doxorubicin, E–C coupling, ryanodine receptors, pachymic acid, losartan

## Abstract

Defects in cardiac contractility and heart failure (HF) are common following doxorubicin (DOX) administration. Different miRs play a role in HF, and their targeting was suggested as a promising therapy. We aimed to target miR-24, a suppressor upstream of junctophilin-2 (JP-2), which is required to affix the sarcoplasmic reticulum to T-tubules, and hence the release of Ca^2+^ in excitation–contraction coupling using pachymic acid (PA) and/or losartan (LN). HF was induced with DOX (3.5 mg/kg, i.p., six doses, twice weekly) in 24 rats. PA and LN (10 mg/kg, daily) were administered orally for four weeks starting the next day of the last DOX dose. Echocardiography, left ventricle (LV) biochemical and histological assessment and electron microscopy were conducted. DOX increased serum BNP, HW/TL, HW/BW, mitochondrial number/size and LV expression of miR-24 but decreased EF, cardiomyocyte fiber diameter, LV content of JP-2 and ryanodine receptors-2 (RyR2). Treatment with either PA or LN reversed these changes. Combined PA + LN attained better results than monotherapies. In conclusion, HF progression following DOX administration can be prevented or even delayed by targeting miR-24 and its downstream JP-2. Our results, therefore, suggest the possibility of using PA alone or as an adjuvant therapy with LN to attain better management of HF patients, especially those who developed tolerance toward LN.

## 1. Introduction

Heart failure (HF) presents a global health burden, at all healthcare levels. It represents the leading cause of hospitalization worldwide, where it affects more than 26 million cases. Despite improved survival, HF prognosis is poor, and mortality rates from HF remain about 17–45% within one year of diagnosis and 45–60% within five years of diagnosis [1,2]. Additionally, most heart diseases can eventually end with HF [1,2], and some drugs may induce HF as a serious side effect, such as the anticarcinogenic drug, doxorubicin [3].

The pathological mechanisms implicated in HF and cardiac remodeling involve complex cellular and molecular signaling cascades, among which are excitation-contraction (E–C) coupling defects [4,5]. E–C coupling represents an important process that governs the contractile strength of cardiomyocytes, where intracellular Ca^2+^ represents a critical mediator. The influx of Ca^2+^ through l-type calcium channels (LCCs) in the cell membrane, including transverse tubules (TTs), activates ryanodine receptors (RyRs) in the junctional sarcoplasmic reticulum (SR) to further release Ca^2+^ in a process called Ca^2+^-induced Ca^2+^ release (CICR) [4]. For normal E–C coupling, a membrane-binding protein, junctophilin-2 (JP-2), is required to affix the SR to TTs. It maintains a 12–15 nm gap between the sarcolemma and the SR membranes in the cardiac dyad ultrastructure, which is required for effective CICR and Ca^2+^ signaling [5]. In HF, the influx of Ca^2+^ from LCCs is not sufficient to induce adequate Ca^2+^ release from RyRs and hence defects in CICR and diminished cardiomyocyte contractility. Moreover, the expression of JP-2 is reduced in failing heart [5,6]. Sarcoplasmic/endoplasmic reticulum Ca^2+^-ATPase 2 (SERCA2), a major Ca^2+^ transport protein in SR, is known to be downregulated in HF. A recent study suggested the involvement of SERCA2a in ameliorating TT remodeling via the calpain/JP-2 pathway and hence cardiac contractility in myocardial ischemia/reperfusion mice [7].

MicroRNAs (miRs)—small, noncoding RNAs of 21–25 nucleotides—are involved in post-transcriptional regulation of gene expression. These are of importance in normal development, as well as various pathological conditions. Most miRs are gene suppressors, while few are activators. Unlike RNA, miRs are stable, extracellular circulating molecules. They have attracted research investigations as potential diagnostic markers and therapeutic targets in various pathological conditions, including HF. Of these miRs, miR-24 is expressed in cardiomyocytes. Similar to other miRs, it can exert different, or even opposite, functions depending on cell type [8,9,10,11]. Even in cardiomyocytes, miR-24 has different/opposite regulatory functions depending on the type of induced injury. In acute injury, miR-24 was suppressed, where its upregulation promoted cell survival and possessed antiapoptotic and antifibrotic effects [12,13]. On the contrary, chronic injuries upregulated miR24 in the heart [8,14,15]. Moreover, serum and cardiac expression of miR-24 was found to be upregulated in atrial fibrillation [16] and heart failure patients [8,17]. Of note, miR24 is an upstream suppressor of JP-2 [6,15,17]; therefore, suppressing miR24 and hence preserving cardiac JP-2 could be a suitable therapeutic strategy in HF.

The management of HF is complex, as it uses different drug categories that can produce an improvement. However, the dose tolerance, toxicity of some of these drugs and cost concerns necessitate the search for alternative or even adjuvant therapeutics for HF. Losartan is an angiotensin receptor type-1 antagonist used in this study as a reference drug for the management of HF.

Pachymic acid (PA; 3-*O*-acetyltumulosic acid) is a natural, lanostrane-type triterpenoid from *Poria cocos*. It has been reported to possess anticarcinogenic, anti-inflammatory and antioxidant effects [18,19,20,21]. The regulation of E–C coupling in doxorubicin (DOX)-induced HF by PA has not been studied yet.

The current study, therefore, aims to examine the possible therapeutic manipulation of DOX-induced HF by PA and to investigate the effect on miR-24 and downstream JP-2 expression as a novel molecular mechanism for E–C coupling regulation against heart failure.

## 2. Results

### 2.1. Cardiac Hypertrophy and Function

**Figure 1:**
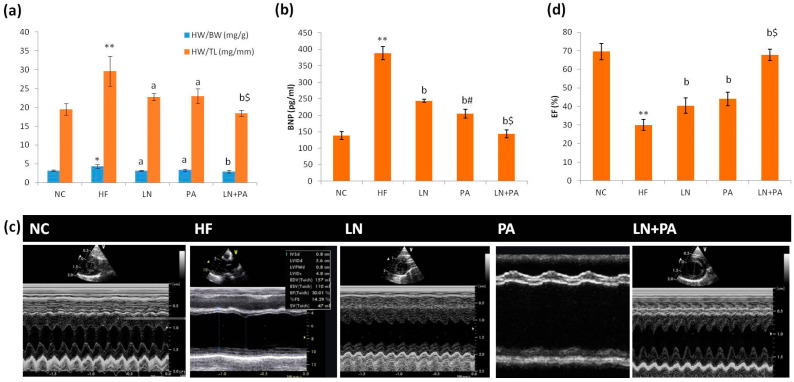
Cardiac weight indices and function in rats with HF. HW/BW and HW/TL (**a**), serum BNP (**b**), echocardiography M-mode tracings through the left ventricle (**c**) and EF (**d**). HF was induced with doxorubicin (3.5 mg/kg, i.p., twice weekly for 3 weeks) and rats were treated with PA (10 mg/kg), LN (10 mg/kg) and their combination (PA + LN) orally and daily for 4 weeks. Results were expressed as mean ± SD (*n* = 6). * *p* < 0.01 and ** *p* < 0.001 from the NC group. ^a^ *p* < 0.01 and ^b^ *p* < 0.001 from the HF group. ^#^ *p* < 0.001 from the LN group. ^$^ *p* < 0.001 from both LN and PA groups. *Abbreviation*: HW/BW, heart weight/body weight; HW/TL, heart weight/tibial length; BNP, brain natriuretic peptide; EF, ejection fraction.

Cardiac hypertrophy was evident in the HF group, which revealed increased HW/BW (*p* < 0.01) and HW/TL (*p* < 0.001) compared to the NC group. Treatment with LN or PA reduced both indices (*p* < 0.01) compared to the HF group. Adding PA to LN further improved these two indices (*p* < 0.001) compared to the HF group, and only HW/TL was lower in the PA + LN group than individual treatments (*p* < 0.001), as shown in Figure 1.

Rats in the HF group had markedly increased serum BNP (*p* < 0.001) compared to rats in the NC group. Treatment with LN, PA or their combination (LN + PA) lowered serum BNP (*p* < 0.001) compared to the HF group. The effect of PA was better than that of LN (*p* < 0.001), and their combination was the best. Only combined (LN+ PA) treatment returned BNP to normal values (Figure 1).

Echocardiography M-mode tracings through the LV of rats from the NC group showed normal systolic function by eyeballing. Rats from the HF group showed marked impairment in systolic function (dilated cardiomyopathy with EF 30 ± 3% by M-mode; *p* < 0.001 compared to NC rats). Treatment with either LN or PA revealed mild improvement in systolic function (EF of 40 ± 4% and 44 ± 4% by eyeballing, respectively) at *p* < 0.001 compared to HF rats. Rats from the LN + PA group showed marked improvement in systolic function (with EF 67 ± 3%; *p* < 0.001 compared to HF rats). LN + PA was better than individual treatments (*p* < 0.001), where only LN + PA treatment returned EF values to normal values, as shown in Figure 1.

### 2.2. Cardiac Architecture and Ultrastructure

**Figure 2:**
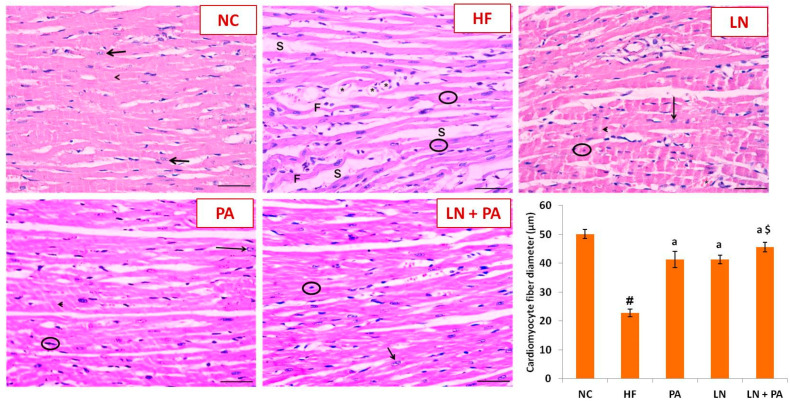
Photomicrographs of sections in the left ventricular myocardium from different experimental groups stained with H&E. HF was induced with doxorubicin (3.5 mg/kg, i.p., twice weekly for 3 weeks) and rats were treated with PA (10 mg/kg), LN (10 mg/kg) and their combination (PA + LN) orally and daily for 4 weeks. Normal control rat (NC) showing cardiomyocytes with acidophilic cytoplasm and striations with central bright nuclei (arrow). The intercalated disc was also seen (arrowhead). The left ventricular myocardium from rats in the HF group showed cardiomyocytes with darkly stained pyknotic nuclei (circle). Cardiomyocyte fibers (F) appear twisted and asymmetrical with loss of the striated appearance of the cardiac fibers. Wide interstitial spaces (S) were noticed among cardiac muscle fibers. Fat cell infiltration was seen between muscle fibers (*). Separated distorted muscle fibers were seen in the interstitium. Left ventricle from rats treated with LN, PA or LN + PA showed almost the same histological structure. Some cardiomyocytes presented with bright vesicular nuclei (arrow), and others had darkly stained nuclei (circle). Intercalated discs were present between the fibers, and cytoplasmic striations were noticed (arrowhead), scale bar 20 μm. The diameter of cardiomyocyte fibers was analyzed in 18 different fields from each group, and data were presented as mean ± SD. ^#^ significant from NC at *p* < 0.001, ^a^ significant from HF at *p* < 0.001 and ^$^ significant from both LN and PA at *p* < 0.001.

Examination of H&E-stained sections of the LV myocardium is shown in Figure 2. Sections from NC rats revealed cardiac muscle fibers with transverse and longitudinal directions. They appeared with acidophilic cytoplasm and striations with oval central vesicular nuclei. The intercalated disc was also seen between the cardiac myofibrils. The ventricular myocardium of rats from the HF group revealed irregularly separated cardiac myocytes with darkly stained nuclei. Loss of the characteristic striated architecture specific to cardiac muscle fibers, along with wide spaces between fragmented myofibrils, were observed. Rats treated with LN, PA and their combination revealed cardiac muscle fibers with minimal spaces in-between. Some of their nuclei were bright, while others appeared dark. Cardiomyocytes appeared striated with the intercalated disc between them. Mild dilated blood vessels were also seen between the cardiac fibers. Cardiomyocyte fiber diameter was significantly decreased in the HF group compared to control littermates (*p* < 0.001). The use of either PA or LN improved the cardiac fiber diameter (*p* < 0.001) compared to the HF group. The maximum response was achieved by the use of combined LN and PA, which was better than individual treatments (*p* < 0.001).

**Figure 3:**
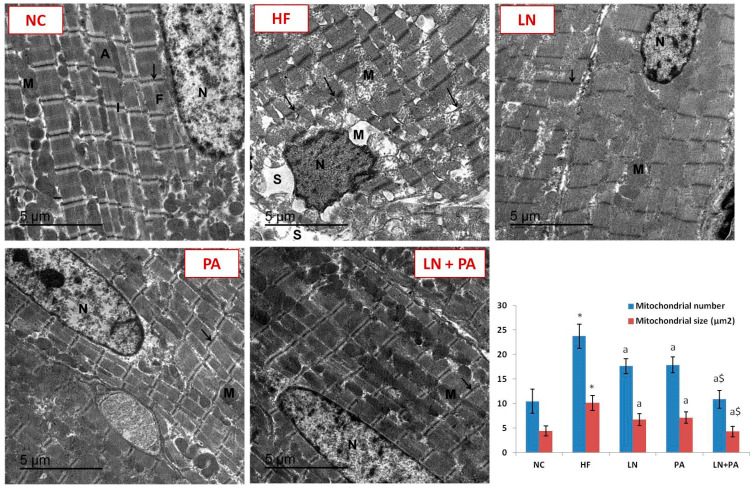
TEM photomicrograph of ultrathin sections in the left ventricular myocardium from different experimental groups. HF was induced with doxorubicin (3.5 mg/kg, i.p., twice weekly for 3 weeks) and rats were treated with PA (10 mg/kg), LN (10 mg/kg) and their combination (PA + LN) orally and daily for 4 weeks. Normal control rat (NC) showed a cardiomyocyte with a euchromatic nucleus. Myofibrils were seen with mitochondrial rows in-between them. Myofibrils presented with alternating light and dark bands. Z lines appeared as intersecting light bands. The left ventricular myocardium from rats in the HF group showed a dark nucleus and peripheral heterochromatin. Swollen mitochondria with ruptured cristae were detected in-between the fragmented myofibrils. Varied spaces between split myofibrils were noticed. The left ventricle from rats treated with LN and PA showed a cardiomyocyte with a heterochromatic nucleus. Some myofibrils were well structured, while others were disordered. Disordered mitochondria were noticed. Sections from the LN + PA group showed cardiomyocytes with a euchromatic nucleus with partial peripheral heterochromatin deposition. Cardiac myofibrils were observed with mitochondrial rows lying between them. Mitochondria were identified with their morphology. Their number and size (μm^2^) were analyzed in 20 different random fields from each group, and data were presented as mean ± SD. * significant from NC at *p* < 0.001, ^a^ significant from HF at *p* < 0.001 and ^$^ significant from both LN and PA at *p* < 0.05. Abbreviations: nucleus, N; mitochondria, M; myofibrils, F; Z lines, arrow; light bands, I; dark bands, A; space, S. Scale bar: 5 μm.

On the ultrastructural level, ultrathin sections from the LV of the NC group revealed a cardiomyocyte with a euchromatic nucleus. Myofibrils were seen with mitochondrial rows in-between them. Myofibrils presented with alternating light and dark bands. Z lines appeared as intersecting light bands. The HF group presented with a dark nucleus and peripheral heterochromatin. Swollen mitochondria with ruptured cristae were detected in-between the fragmented myofibrils. Varied spaces between split myofibrils were noticed. HF rats treated with either LN or PA displayed a cardiomyocyte with a heterochromatic nucleus. Some myofibrils were well structured, while others were disordered. Disordered mitochondria were noticed. HF rats treated with both LN and PA showed cardiomyocytes with a euchromatic nucleus with partial peripheral heterochromatin deposition. Cardiac myofibrils were observed with mitochondrial rows lying between them. Mitochondrial number and size increased markedly in cardiomyocytes from HF rats compared to cardiomyocytes from NC rats (*p* < 0.001). LN, PA or their combination decreased the size and number of mitochondria compared to untreated HF rats (*p* < 0.001) (Figure 3).

**Figure 4:**
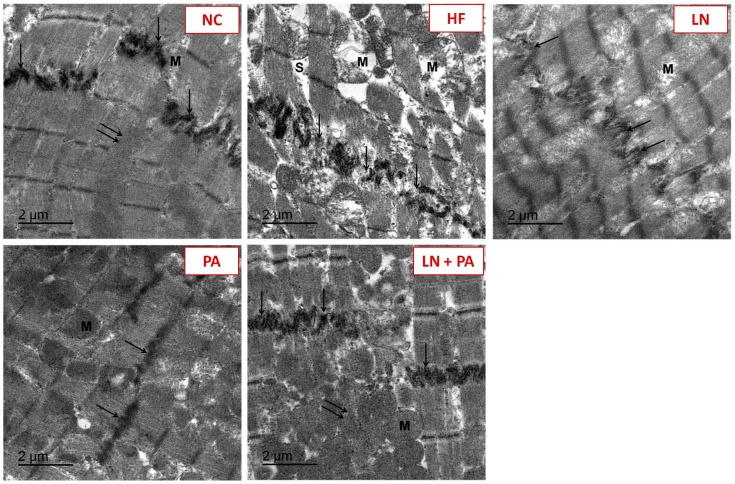
TEM images of intercalated discs in-between cardiac myofibrils in different experimental groups. HF was induced with doxorubicin (3.5 mg/kg, i.p., twice weekly for 3 weeks) and rats were treated with PA (10 mg/kg), LN (10 mg/kg) and their combination (PA + LN) orally and daily for 4 weeks. NC rats showed well-ordered intercalated discs and glycogen particles in-between cardiac myofibrils. HF rats showed disordered intercalated discs between myofibrils. Some mitochondria are swollen with disturbed cristae and membrane. Increased spaces between cardiac myofibrils are also seen. Both LN- and PA-treated groups showed partially organized intercalated discs with swollen mitochondria. LN + PA-treated rats showed near to normal intercalated disc and glycogen particles between myofibrils. Abbreviations: intercalated discs, arrow; glycogen particles, double arrows; mitochondria, M; spaces, S. Scale bar: 2 μm.

The intercalated discs in-between cardiac cells examined on the ultrastructural level are presented in Figure 4. NC rats showed well-ordered intercalated discs and glycogen particles in-between cardiac myofibrils. However, HF rats showed disordered intercalated discs between myofibrils. Some mitochondria were swollen with disturbed cristae and membrane. Increased spaces between cardiac myofibrils were also seen. HF rats treated with either LN or PA showed partially organized intercalated discs with swollen mitochondria. Rats that received combined LN and PA treatment showed near to normal intercalated discs and glycogen particles between myofibrils.

### 2.3. miR 24 and LCC-RyR Signaling

**Figure 5:**
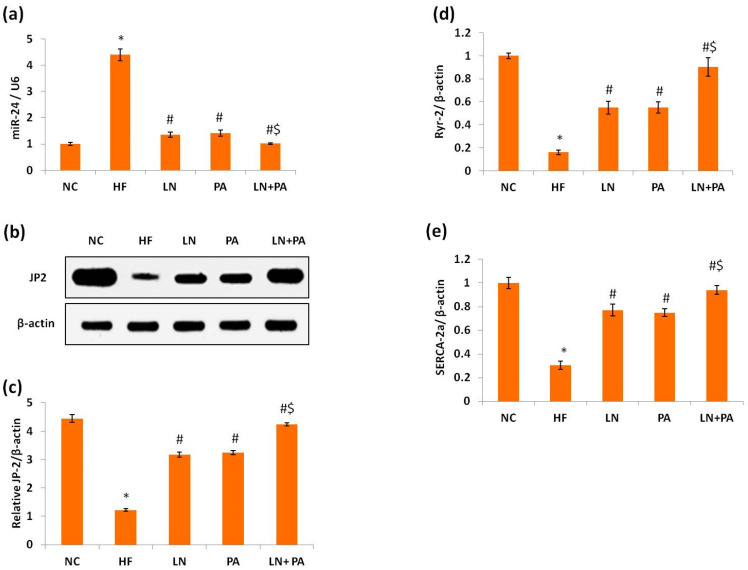
miR24 and LCC-RyR signaling in the left ventricle of rats with heart failure.HF was induced with doxorubicin (3.5 mg/kg, i.p., twice weekly for 3 weeks) and rats were treated with PA (10 mg/kg), LN (10 mg/kg) and their combination (PA + LN) orally and daily for 4 weeks. Left ventricular expression of miR-24 (**a**), JP-2 (**b**) representative Western blotting of JP-2 and β-actin, (**c**) quantitation of JP-2 protein expression normalized to β-actin housekeeping protein and mRNA expression of (**d**) RyR-2 and (**e**) SERCA2a. Data are mean ± SD, *n* = 6. * *p* < 0.001 compared to the NC group, ^#^ *p* < 0.001 compared to the HF group, ^$^ *p* < 0.001 compared to both LN and PA groups. Abbreviations: junctophilin-2, JP-2; ryanodine receptor 2, RyR2; sarcoplasmic/endoplasmic reticulum Ca^2+^-ATPase 2, SERCA2.

The expression of miR24 was upregulated by 4.4 folds in the LV of rats in the HF group compared to rats in the NC group (*p* < 0.001). On the other hand, these rats showed downregulation in mRNA expression of both RyR-2 (6.7 folds) and SERCA-2a (2.98 folds) compared to the NC group (*p* < 0.001). The protein expression of JP-2 was decreased (3.6 folds) in the LV of rats in the HF group (*p* < 0.001) compared to NC rats (Figure 5).

Treatment with PA, LN or PA + LN impeded the upregulation in miR24 and the downregulation in mRNA expression of RyR-2 and SERCA-2a genes and the protein expression of JP-2 compared to the HF group (*p* < 0.001). PA and LS groups showed comparable results on these parameters. Combined PA + LN treatment was the best among all treatments (*p* < 0.001), as shown in Figure 5.

To further confirm the relationship between miR24 targeting by the treatment we used in this study and the regulation of HF in our DOX model, a correlation study was conducted, and the results are illustrated in Table 1. miR24 was strongly correlated with serum BNP, HW/TL and mitochondrial number and size but negatively correlated with cardiomyocyte diameter, JP-2, SERCA-2a and RyR2 (*p* < 0.001, *n* = 30). The expression of JP-2 was also shown to be strongly correlated with the mRNA expression of both RyR-2 and SERCA-2a genes (*p* < 0.001, *n* = 30).

## 3. Discussion

To study HF with nonischemic etiology, we utilized a DOX-induced rat model, which waswidely reported [22,23]. Dysregulation of E–C coupling and reduced contractility as a cause of HF was previously reported in this model [15]. In clinical settings, the use of DOX as an anticancer agent is associated with cardiotoxic effects and HF with poor prognosis. Both acute and chronic toxicities have been reported. With cumulative DOX doses especially, adult survivors with childhood cancer developed delayed cardiomyopathy and HF four to twenty years following DOX treatment that was dose dependent. Short-term use or even dose reduction to lower this cardiotoxicity might disrupt anticarcinogenic activity [3]. Using a DOX-induced HF rat model, we reported, for the first time, the therapeutic potential of PA in HF and proved its modulatory effect on myocardial contractility by preserving cardiac JP-2 through suppression of miR-24.

In this study, HF was confirmed to be induced by DOX, as indicated by increased serum BNP level, along with reduced EF% and FS%. These changes associated with DOX administration show ventricular dysfunction [24]. Moreover, cardiac hypertrophic dilatation was also induced by DOX, as indicated by increased heart weight indices. These results are in accordance with previous studies [15,24,25].

The LV architecture of HF rats also revealed cardiomyocyte fibers with reduced diameter and increased spaces between cardiac fibers, which lost their characteristic striation, along with collagen deposition (data not shown) in-between these fibers. These changes in cardiac muscle fibers may explain the changes in mechanics and hence cardiac force/contractility [26]. We, therefore, looked closer at investigating the LV cardiomyocyte ultrastructure using TEM, which revealed an increased number and size of swollen mitochondria with disturbed cristae and membrane, along with disordered intercalated discs between myofibrils in HF rats. Our results are in accordance with previous studies [15,27]. The intercalated discs play an important role in the transmission of action potentials and Ca^2+^ during muscle contraction, where the coordination of cardiomyocytes’ contraction is controlled through the gap junctions of intercalated discs [28]. With these changes in intercalated discs, contractility dysregulation and defects, E–C coupling is the proposed cause of HF in our DOX model, namely the uncoupling between TT and SR [15,17].

In this study, to investigate the uncoupling between TT and SR as a molecular mechanism underlying dysregulated contractility in DOX-induced HF, we investigated the key regulator of TT–SR junctions, JP-2. The latter was extensively reported to be downregulated or mislocalized in different animal models of HF, including DOX-induced HF [15,17]. We reported downregulation of JP-2 in the LV tissue of our HF rat model. JP-2 is a structural protein that is considered the key regulator of TT–SR junctions. It spans the junctional distance in the subcellular domains, coupling TTs and RyRs to further release Ca^2+^ in the CICR process [4]. The downregulation of JP-2, therefore, resulted in uncoupling between TTs and RyRs [29]. Li and colleagues reported shortened TT–SR junctions and increased cleft distance of TT–SR junctions in DOX-induced HF rats [15]. As a result, the influx of Ca^2+^ from LCCs was not sufficient to induce adequate Ca^2+^ release from RyRs and hence defects in CICR and diminished cardiomyocytes contractility [4,17,29], as seen in our HF rats, which exhibited reduced EF. Of note, the embryonic myocytes’ JP2 deficiency was reported to be associated with defective cardiac dyads, segmentation in SR with T-tubule uncoupling, along with defective intracellular Ca^2+^ transients [30]. Accordingly, the gene expression of both RyR-2 and SERCA-2a in our HF rats was downregulated. These results are in accordance with [7,17,31]. On the contrary, Li et al. reported no change in the mRNA expression of RyR-2 in their HF model [15].

Since miR24 was previously reported to regulate JP-2 in cardiomyocytes, acting as an upstream suppressor of JP-2 [6,15,17] and upregulated in heart failure patients [8,17], we measured its expression, which was highly upregulated in our HF rats. The overexpression of miR-24 in rat ventricular myocytes, therefore, suppressed JP-2 [6], which explains the downregulated JP-2 protein expression. On the other hand, the suppression of miR-24 by using a specific antagomir in a chronic mouse model of pressure-overload hypertrophy prevented the transition toward decompensated hypertrophy by stabilizing JP-2 expression and preserving the ultrastructure of TTs–SR junctions [14]. MiR-24 was, therefore, suggested as a therapeutic target to regulate JP-2 expression, CICR response and cardiac contractility in failing heart [6,17].

In this context, we utilized LN- and PA-targeting miR-24 to reverse the changes induced by DOX. PA significantly suppressed miR-24 expression with concomitant upregulation of JP-2 protein expression in the LV compared to HF rats. This restored the TT–SR junction’s ultrastructure and hence improved CICR-regulating cardiac contractility, which, in turn, explains the improvement in cardiac function shown by increased EF% and BNP levels in our treated rats. We further obtained a strong, positive correlation between the LV expression of miR-24 and serum BNP. On the other hand, the LV expression of miR-24 was negatively correlated with LV expression of JP-2, mRNA expression of RyR-2 and SERCA-2a and EF%. In other words, the deterioration toward HF following the administration of DOX was prevented by either PA or LN. Combined LN and PA revealed better results compared to monotherapies in all studied parameters.

In conclusion, heart failure progression following the administration of doxorubicin can be prevented or even delayed by targeting miR-24 and its downstream JP-2. The in vivo suppression of miR-24 using either PA or LN stabilized JP-2 expression, which restored the coordination between TTs and junctional SRs, preserving deteriorated E–C coupling following the administration of DOX. Our results, therefore, suggested the possibility of using PA alone or as an adjuvant therapy with LN to attain better management of HF patients, particularly those who received the anticancer drug doxorubicin and those who developed tolerance toward LN. Further preclinical and clinical studies are highly recommended to further confirm the applicability of these results in clinical practice.

## 4. Materials and Methods

### 4.1. Drugs

Doxorubicin (Adricin^®^) was purchased from Hikma Pharmaceuticals, Giza, Egypt. Losartan (Cozaar^®^) was purchased from MSD, New Cairo, Egypt. PA was purchased from CNLab Nutrition, Asian Group, Shaanxi, China.

### 4.2. Animals and Experimental Design

Thirty male albino rats (180 ± 20 g) were purchased from the animal unit at the Faculty of Veterinary Medicine, Zagazig University, Zagazig, Egypt. Rats were housed in stainless-steel cages under controlled environmental conditions and allowed free access to rodent pellet chow and drinking water.

After one week of acclimatization, HF was induced in 24 rats by doxorubicin (3.5 mg/kg; i.p., twice weekly) for 3 weeks [22]. After the last dose of DOX, rats were subdivided into four groups: the HF group received drug vehicle, the PA group received PA (10 mg/kg) orally and daily for 4 weeks [32], LN (10 mg/kg) orally and daily for 4 weeks [33] and the combined PA + LN group received PA and LN for four weeks as previously described. The remaining six rats were used as a normal control (NC) group.

The experimental design and all experimental procedures followed the ethical guidelines for investigations in laboratory animals and were approved by the Institutional Animal Care and Usage Committee at Zagazig University (ZU-IACUC/3/F/147/2019).

### 4.3. Echocardiography

Echocardiography for rats (received ketamine/xylazine (50/1 mg/kg) anesthesia) was carried out prior to scarification [34]. M-mode images of LV were recorded to assess the function of the left ventricle (LV) expressed as ejection fraction (EF). Measurements were performed according to ordinary American Society of Echocardiography guidelines [35].

### 4.4. Sampling

At the end of the experiment, rats were anesthetized, blood was collected to separate serum and then rats were euthanized by decapitation. The heart was then removed, rinsed with ice-cooled saline, dried and weighed. The LV was dissected, part of which was flash-frozen in liquid nitrogen and kept at −80 °C for further biochemical analyses. Another portion was kept in 4% formol saline and processed for histological studies. Small pieces (1 mm^3^) from LV were kept overnight at 4 °C in a mixture of 2% glutaraldehyde and 4% paraformaldehyde prepared in 0.1 M sodium cacodylate buffer (pH 7.2) then processed for transmission electron microscopy (TEM).

### 4.5. Biochemical Analyses

#### 4.5.1. ELISA of Serum BNP

Serum brain natriuretic peptide (BNP) was measured according to Maisel et al. [36] using the ELISA kit provided by Abnova (Taoyuan City 320, Taiwan).

#### 4.5.2. RT-PCR Cardiac Expression of miR-24-3, RYR-2 and SERCA-2a

Total RNA was extracted from LV using Direct-zol™ RNA Miniprep Plus TRIzol^®^ Inc. RNA Out (Cat. No.: R2071), supplied by Zymo Research Corporation, Tustin, CA, USA). RNA was reverse-transcribed using the Invitrogen SuperScript™ IV One-Step RT-PCR System kit (Cat. No.: 12594100) supplied by Thermo Fisher Scientific, Waltham, MA, USA. The sequences of primers for miR-24-3p, RyR2, SERCA-2a and U6 and β-actin housekeeping genes are shown in Table 2. The thermal cycler RT-PCR protocol was 1 cycle (10 min at 45 °C; reverse transcription), 1 cycle (2 min at 98 °C; RT inactivation and initial denaturation) and 40 cycles (10 s at 98 °C, 10 s at 55 °C and 30 s at 72 °C; amplification). Applied Biosystems StepOne™ Real-Time PCR system (Applied Biosystem, Waltham, MA, USA) was utilized. 2^−∆∆Ct^ method was used to calculate the relative gene expression using the housekeeping gene U6 for miR-24 and β-actin for other genes.

#### 4.5.3. WB-Cardiac JP-2

Total protein was extracted from LV and quantified using the ReadyPrep™ Protein Extraction Kit (Total Protein) (Cat. No.: 1632086) and Bradford Protein Assay Kit (SK3031) supplied by Bio-Rad Laboratories (Dubai, United Arab Emirates) and Bio Basic Inc. (Markham, ON, Canada), respectively, following the manufacturers’ instructions. Protein from samples (20 μg) was diluted with an equal volume of sample buffer (2× Laemmli buffer containing 4% SDS, 10% 2-mercaptoethanol, 20% glycerol, 0.004% bromophenol blue and 0.125 M Tris HCl, pH 6.8). The mixture was boiled for 5 min at 95 °C (protein denaturation). Electrophoresis was performed using the TGX Stain-Free™ FastCast™ Acrylamide Kit (Cat. No.: 161-0181) provided by Bio-Rad Laboratories (Dubai, United Arab Emirates), according to the manufacture’s instructions. The separated bands were transferred into the PVDF membrane using 1× transfer buffer (25 mM Tris and 190 mM glycine and 20% methanol) at 25 V for 7 min. The membrane was blocked with 3% bovine serum albumin (BSA) in tris-buffered saline containing 0.1% Tween 20 (TBST) at room temperature for 1 h. Membranes were incubated overnight at 4 °C with TBST-diluted primary antibodies of JP-2 antibody (sc-377086) and β-actin (sc-47778) purchased from Santa Cruz Biotechnology, Inc. (Heidelberg, Germany). The blots were washed 3 times with TBST and then incubated with HRP-conjugated goat IgG monoclonal secondary antibody (Novus Biologicals LLC, Centennial, CO, USA) for 1 h at room temperature. The blots were washed 3 times with TBST, and the chemiluminescent substrate (Clarity™ Western ECL substrate, Bio-Rad, Cat. No.: 170-5060) was applied to the blots. The chemiluminescent signals were captured using a CCD camera-based imager. ImageJ software was used to measure the band intensity of JP-2 and expressed relative to that of β-actin.

### 4.6. Histopathology

Routine histological procedures were followed to stain LV tissue. Briefly, formalin-fixed LV tissue was dehydrated in ascending grades of alcohol, embedded in paraffin, sectioned into 5 µm thick sections, dewaxed in xylol, rehydrated in descending grades of alcohol and stained with hematoxylin and eosin. Three random fields were captured per rat, and photographs were analyzed. The diameter of cardiomyocyte fibers was measured in H&E-stained sections using the Leica Qwin 500 image analyzer computer system (Leica Microsystems GmbH, Wetzlar, Germany) in the image analysis unit in the Department of Pathology, Faculty of Dentistry, Cairo University, Egypt.

### 4.7. Transmission Electron Microscopy (TEM)

Further fixation of LV tissue was performed in 2% osmium tetroxide before tissue dehydration in ascending grades of alcohol, cleared in propylene oxide and impregnated in resin to make hard capsules ready for sectioning. The Leica Ultracut UCT microtome (Leica Microsystems, Germany) was used to make 150 nm semithin sections, which were ultrasectioned into 80 nm thin sections mounted on a copper grid. Staining was performed by uranyl acetate, and lead citrate and sections were then visualized at 160 kV using a JEOL TEM (JEM-2100, Tokyo, Japan) at the Electron Microscope Unit, Mansoura University, Egypt. Twenty random fields were captured per group. Microphotographs were analyzed to measure mitochondrial size (average cross-sectional diameter) and mitochondrial numbers using the Leica Qwin 500 image analyzer computer system (Leica Microsystems GmbH, Wetzlar, Germany) in the image analysis unit in the Department of Pathology, Faculty of Dentistry, Cairo University, Egypt.

### 4.8. Statistical Analysis

Results were expressed as mean ± SD, and the statistical difference between groups was analyzed using one-way analysis of variance (ANOVA), followed by Tukey’s post-hoc test, taking *p* < 0.05 as statistically significant. GraphPad Prism 7 software (Graph Pad, San Diego, CA, USA) was used.

## Figures and Tables

**Table 1:** Correlation between studied parameters in HF rats treated with LN, PA and their combination.

Gene	miR24	JP2	SERCA-2a	RyR2
**HW/BW**	0.81	−0.80	—	—
**HW/TL**	0.82	−0.88	—	—
**JP2**	−0.94	—	—	—
**SERCA-2a**	−0.95	0.98	—	—
**RyR2**	−0.85	0.97	0.94	—
**BNP**	0.93	−0.98	−0.97	−0.94

*n* = 30 and all *p* < 0.001. **Abbreviations**: heart weight/body weight, HW/BW; heart weight/tibial length, HW/TL; brain natriuretic peptide, BNP; junctophilin-2, JP-2; ryanodine receptor 2, RyR2; sarcoplasmic/endoplasmic reticulum Ca^2+^-ATPase-2a, SERCA-2a.

**Table 2:** Primer sequence.

Gene	Forward Primer (5′-3′)	Reverse Primer (5′-3′)
miR-24-3p	CTCTGGCTCAGTTCAGCAG	GAATACCTCGGACCCTGC
RyR2	TGCTGCGAGCCGGG	TGGCGGTGGCGTAGGA
SERCA-2a	CTGGCCGACGACAACTTCTC	TGAGGTAGCGGATGAACTGCTT
U6	CTCGCTTCGGCAGCACATA	AACGATTCACGAATTTGCGT
β-actin	CTAAGGCCAACCGTGAAAAG	GCCTGGATGGCTACGTACA

## Data Availability

The data presented in this study are available on request from the corresponding author.
